# Dissociable Behavioral and Neural Correlates for Target-Changing and Conforming Behaviors in Interpersonal Aggression

**DOI:** 10.1523/ENEURO.0273-19.2020

**Published:** 2020-06-01

**Authors:** Kyosuke Takami, Masahiko Haruno

**Affiliations:** 1 Center for Information and Neural Networks, National Institute of Information and Communications Technology, Suita, Osaka 565-0871, Japan; 2 Graduate School of Frontier Biosciences, Osaka University, Suita, Osaka 565-0871, Japan

**Keywords:** bullying, dACC-insula, interpersonal aggression, resource control theory, resting-state fMRI, target changing

## Abstract

Actors in interpersonal aggression such as bullies change their targets frequently, but the underlying behavioral and neural mechanisms are unknown. Here, using the catch-ball task we recently developed to examine human interpersonal aggression, we found target-changing and conforming to other participants’ aggression are major driving forces of increased aggression (i.e., throwing strong balls). We also found that target-changing was correlated with a participant’s extraversion, consistent with a bistrategic view, in which both prosocial and coercive motivations drive interpersonal aggression. In contrast, conforming to others was correlated with social anxiety. In addition, questionnaires about participants’ past experiences of bullying suggested that target-changers and conformers were predominantly bullies and victims in the past. An analysis of resting-state functional magnetic resonance imaging (fMRI) revealed that functional connectivity between the dorsal anterior cingulate cortex (dACC) and insula were correlated with target-changing behavior, while functional connectivity between the amygdala and temporo-parietal junction (TPJ) was correlated with conformity. These results demonstrate that target-changing and conforming behaviors have dissociable behavioral and neural mechanisms and may contribute to real-world interpersonal aggressions differently.

## Significance Statement

Our model-based integration of behaviors in a catch-ball task and resting-state functional magnetic resonance imaging (fMRI) data demonstrate that target-changing and conforming behaviors have dissociable behavioral and neural mechanisms and contribute to real-world interpersonal aggressions differently.

## Introduction

In many communities, interpersonal aggression such as bullying has become an increasingly serious problem (UNESCO, 2019). As a key instance of interpersonal aggression, recent studies of bullying have emphasized the importance of target-changing. One reported that bullies can victimize more than one peer and change their targets easily (Chan, 2006), and cohort studies have shown that the number of targets are unstable (Pellegrini and Long, 2002; Ryoo et al., 2015). Furthermore, a recent analysis of social network behavior revealed that bullies tend to switch their victims and refine strategies to access effective targets (Huitsing et al., 2014; van der Ploeg et al., 2020).

The behavioral and neural mechanisms of target-changing behavior can be viewed from an evolutionary perspective (Hawley et al., 2011). Influential resource control theory classifies individuals into types based on their relative usage of prosocial and coercive strategies (Hawley, 2002, 2003; Little et al., 2007). Bullies can use different strategies, appearing as coercive controllers or bistrategic controllers. Bistrategic controllers, who use both prosocial and coercive strategies, tend to be more successful than coercive controllers. They are better integrated into the social group, perceived to be more popular and attractive than the majority of their peers, suggesting a bistrategic approach in bullying may be evolutionally advantageous. One previous study examined the link between bistrategic controllers and bullying by analyzing the content of participants’ chat texts during interactive resource allocation games and finding that the participants who often utter both prosocial and coercive statements are associated with more relational aggression as measured by questionnaires (Mancilla-Caceres et al., 2015). Based on these considerations, we hypothesized that the target-changing in bullying occurs as a bistrategic strategy, because target-changing makes it possible to behave prosocially (i.e., helping a victim and punishing a bully) while continuing the aggression simultaneously. However, there is no behavioral task of interpersonal aggression that can examine such bistrategic behavior and its underlying neural mechanism.

In the context of aggression, several previous studies have successfully measured individual-level aggression or reactive aggression (Bandura et al., 1961; Taylor, 1967) and its neural substrates (Nelson and Trainor, 2007; Rosell and Siever, 2015), but far fewer have focused on group aggression (Meier et al., 2007) such as bullying or peer victimization. These studies mainly used Cyberball, an interactive task that recreates social exclusion situations, and demonstrated that a higher activation of the dorsal anterior cingulate cortex (dACC) and insula in those who are socially rejected or isolated (Eisenberger et al., 2003; Masten et al., 2013; Chester et al., 2014; Vijayakumar et al., 2017; Perino et al., 2019). Although most studies focused on victimization, one exception focused on those who bully (Perino et al., 2019) by scanning adolescents who observed instances of social exclusion and inclusion during Cyberball. The authors reported that the self-reported bullying score was associated with a higher activation of the ventral striatum, amygdala, medial prefrontal cortex, and insula activation, which was identified by contrasting social exclusion and inclusion conditions, linking Cyberball and bullying.

**Figure 1.**
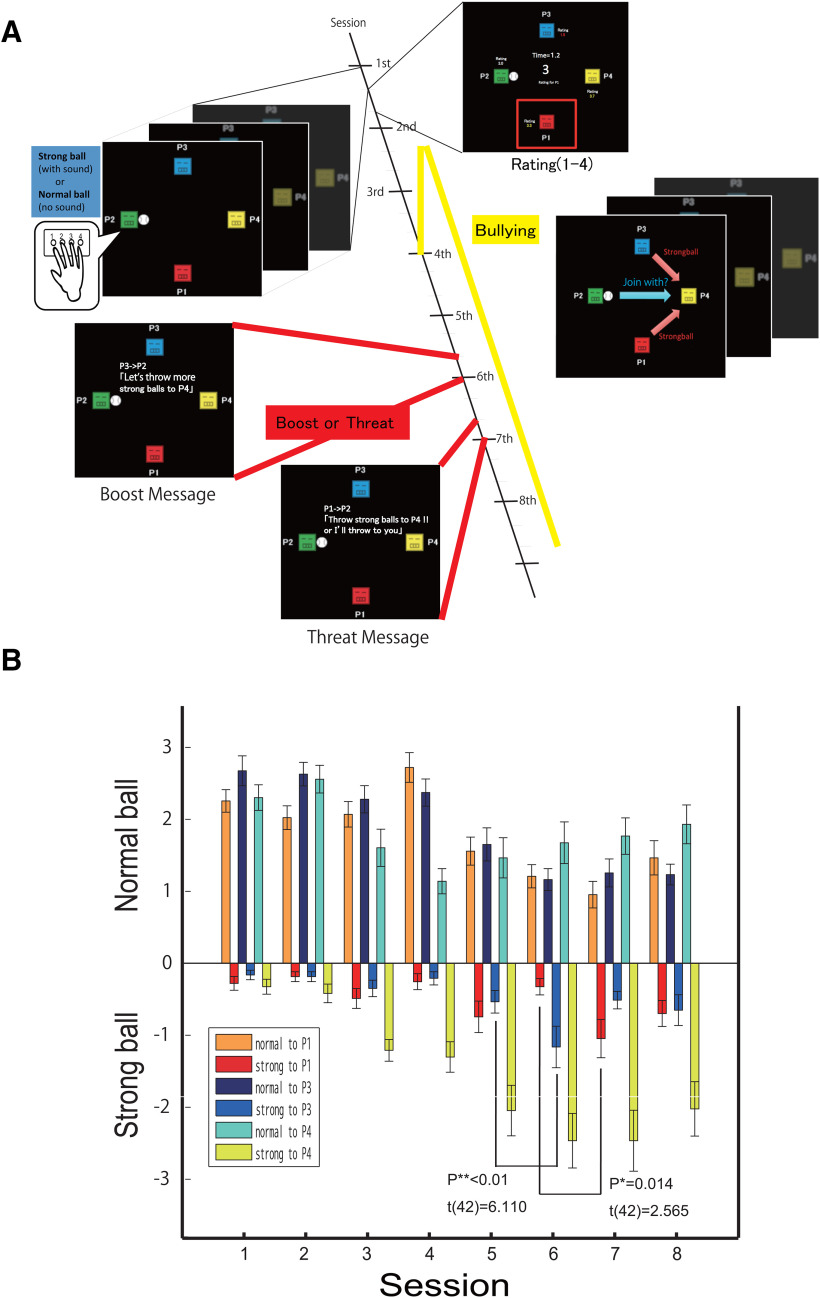
Task overview and player behavior. ***A***, Task overview. Participants play the catch-ball game and are assigned a player number (P1–P4). In reality, all participants are unknowingly assigned P2, and the actions of P1, P3, and P4 are controlled by a computer program. In each session, each player rates the other players (1–4 scale). Participants can send messages to the other players in all sessions. In session 3, P4 begins to throw strong balls with an equal probability to other players. In session 4, P1 starts to throw strong balls to P4 repeatedly. In session 5, P3 starts to throw strong balls with P1 to P4. In session 6, P2 receives the message from P3, “Let’s throw more strong balls to P4.” In session 7, P2 receives a boost or threat message from P1, “Let’s throw more strong balls to P4” or “Throw strong balls to P4 or I’ll throw them to you.” ***B***, Participant behavior and ratings. Bar graphs show the mean ball frequencies (normal or strong) per session. Normal balls are shown in the positive region, and strong balls in the negative region. From session 5 to session 6, the number of strong balls thrown to P3 increased. From session 6 to session 7, the number of strong balls thrown to P1 increased. A detailed task flow is illustrated in Extended Data Figure 1-1.

We previously developed a novel catch-ball task similar to Cyberball to examine how participants behave when others start interpersonal aggression and analyzed resting-state functional magnetic resonance imaging (fMRI) data based on behaviors during the task (Takami and Haruno, 2019). The task required four participants play together on individual desktop computers (Fig. 1*A*). However, unknown to the participants, they were all assigned a single role (P2), and the other three players were preprogrammed computer agents (P1, P3, and P4). These players in turn “threw” balls at one of two strengths (normal and strong). Strong balls were associated with an unpleasant sound that was mildly harmful to the recipient player. In sessions 4 and 5 of the task (eight sessions in total), two players (P1 and P3) started to throw strong balls to one victim player (P4) repeatedly. Each participant threw eight balls in each session (64 balls in total), and this setting enabled us to examine whether the participants (P2) conformed to laboratory interpersonal aggression or not. We reported that each participant’s degree of conformity was correlated with a social anxiety score (and neuroticism score) and not an empathy score, and also that the degree of conformity was correlated with functional connectivity between the amygdala and temporo-parietal junction (TPJ) and between the ventral ACC and posterior cingulate cortex (PCC; Takami and Haruno, 2019).

10.1523/ENEURO.0273-19.2020.f1-1 Extended Data Figure 1-1 Behavior of players other than P2 (supporting Fig. 1). Session 1: P1, P3, and P4 only throw normal balls to other players with equal probability. Session 2: P1 and P3 never throw a ball to P4. P1 and P3 only throw normal balls (blue arrows). Session 3: P4 throws strong balls (orange arrows) to others with equal probability. P1 and P3 throw only normal balls to P2 and P4. Session 4: P1 throws strong balls to P4 with a probability of 80%. Session 5: P1 and P3 throw strong balls to P4 with a probability of 80%. This behavioral pattern continues in subsequent sessions. Download Figure 1-1, EPS file.

Here, we shift our attention to target-changing behavior by focusing on behaviors after session 6, in which a message was sent from P3 to P2 to boost aggression. If P2 decided not to participate in the aggression, a threat message was sent from P1 to P2 before the start of session 7. We observed that after the boosting messages, some participants who conformed to the aggression to P4 changed their target of aggression from P4 to P1 and P3, who had been throwing strong balls at P4. This target-changing behavior may well reflect bistrategic (prosocial and coercive) intention, because it looks to help a victim (or punish a bully) and simultaneously continues the aggression. To examine the underlying behavioral and neural mechanisms, we extended our previous computational model to incorporate target-changing and analyzed resting-state fMRI scans of the same participants.

To increase the validity of our behavioral task, we had the four participants conduct the task so that they can physically observe one another. However, a task-based fMRI experiment separates the one participant in the MRI scanner from the three other participants, reducing the reality of the experimental setting. Therefore, we used resting-state fMRI in the present study in combination with a behavioral experiment (the catch-ball task). We previously found a correlation between resting-state fMRI connectivity and conformity (and social anxiety) using this task. Therefore, we expected that resting-state fMRI would be effective for identifying the neural correlates of the target-changing behavior during the same task.

To link the results obtained from these experiments to real-world interpersonal aggression and the personality traits of the participants, we included a self-report measure of past experiences of bullying, a self-report measure of personality [Big Five Inventory (BFI); John and Srivastava, 1999] and a self-report measure of empathy [Interpersonal Reactivity Index (IRI); Davis, 1983]. We hypothesize that the dACC may play an important role, because this brain structure was revealed to correlate with conformity to interpersonal aggression in our previous study (Takami and Haruno, 2019), with social exclusion in the above catch-ball setting (Kawamoto et al., 2012) and with behavioral switching in foraging tasks (Kolling et al., 2012), which is relevant to bistrategic behavioral choices.

## Materials and Methods

### Participants

Informed consent was obtained from all participants, and the experimental protocol was approved by the ethics committees of National Institute of Information and Communications Technology (NICT). Forty-three male undergraduate and graduate students aged 20–26 (21.6 ± 1.5 years) participated in both the behavioral and resting-state fMRI experiments. For each participant, two experiments were conducted at least 72 h apart. As such, little effect was expected between the two experiments. All participants were males, because males were reported to be more aggressive than females in physical settings such as the catch-ball task (Maccoby and Jacklin, 1974; Mitchell, 1981).

### Behavioral tasks

Four participants were invited into an experiment room together and sat at different desks equipped with a desktop computer and a display. The catch-ball task consisted of eight sessions (Fig. 1). Each player could “throw” a ball on the screen at two different strengths (normal or strong) to any of the other three players. Strong balls were associated with an unpleasant sound that was mildly harmful to the recipient player.

Although we instructed the four participants that they would play the game as different players, all of them were unknowingly assigned the role of player 2 (P2: participant) and played against three computer-programmed players (P1, P3, and P4: computer; see also Extended Data Fig. 1-1). Since the participants did not know any other assigned player number, player anonymity was maintained. In each session, after each participant threw eight balls in total (the condition that ended the session), the four players were asked to rate the other players’ behavior within the session on a scale of 1–4 (1 = bad, 4 = good). They were also permitted to send messages to the other players. On average, each participant threw 20.05 strong balls and 43.90 normal balls in the entire 64 ball throws during the task.

More specifically, the catch-ball game proceeded as follows (see also Fig. 1; Extended Data Fig. 1-1):

Session 1: P1, P3, and P4 throw normal balls to other players with equal probability.

Session 2: P1 and P3 never throw a ball to P4.

Session 3: P4 throws strong balls to other players with equal probability. By contrast, P1 and P3 throw only normal balls.

Session 4: P1 throws only strong balls to P4.

Session 5: P1 and P3 throw only strong balls to P4.

Session 6: Before this session, P2 receives the message from P3: “Let’s throw more strong balls to P4.”

Session 7: If P2 never throws a strong ball to P4 in session 6, he receives the message: “Throw strong balls to P4 or I’ll throw them to you” from P1, and P1 and P3 throw only strong balls to P2 and P4. Otherwise, P2 receives the message: “Let’s throw more strong balls to P4” from P1, and P1 and P3 continue to throw only strong balls to P4.

Session 8: P1 and P3 continue to throw strong balls the same way as in session 7.

We included a postexperiment questionnaire to assess the ecological validity of the task, and ∼90% of the participants accepted the task manipulation as valid (for further details, see Takami and Haruno, 2019).

### Model-based analysis

To analyze each participant’s peer-influenced participation in interpersonal aggression quantitatively, we extended the utility function, U(X_t_), which we used in our previous report (Takami and Haruno 2019), based on normal and strong ball throws to different players in each trial as Equation 1. Note that normal and strong balls can have different meanings depending on the session and target player. For example, after session 3, a normal ball to P4 would help P4, while a normal ball to P1 or P3 may represent indirect aggression to P4.

(1) $$\text{U}\left({\text{X}}_{\text{t}}\right)=\beta 0+\beta \text{1}\;{\text{f}}_{\text{1}}\left({\text{X}}_{\text{t}}\right)+\beta \text{2}\;\text{react}\left(\text{t}\right)\;{\text{f}}_{\text{2}}\left({\text{X}}_{\text{t}}\right)+\beta \text{3 conf}\left(\text{t}\right)\;{\text{f}}_{\text{3}}\left({\text{X}}_{\text{t}}\right)+\beta \text{4}\;\text{message}\left(\text{t}\right)\;{\text{f}}_{\text{4}}\left({\text{X}}_{\text{t}}\right)+\beta \text{5 total}\_\text{strong}\_\text{balls}\left(\text{t}\right)\;{\text{f}}_{\text{5}}\left({\text{X}}_{\text{t}}\right)+\beta \text{6 change}\left(\text{t}\right)\;{\text{f}}_{\text{6}}\left({\text{X}}_{\text{t}}\right),$$

with β0: intercept, β1: baseline propensity for aggression, β2: reactive revenge, β3: conformity to aggression, β4: capitulation to threat, β5: accumulation effect of previous strong balls, and β6: target-change. In addition,

$${\text{f}}_{\text{1}}\left({\text{X}}_{\text{t}}\right)=\{\begin{array}{c} 1:Xt=S1,S3,S4\\ -1:Xt=N1,N3,N4 \end{array}$$

$${\text{f}}_{\text{2}}\left({\text{X}}_{\text{t}}\right)=\{\begin{array}{c} 1:Xt=S4\\ -1:Xt=\left(\text{N}1,\;\text{N}3,\;\text{N}4\right)\;or\;\left(\text{S}1,\;\text{S}3\right) \end{array}$$

$${\text{f}}_{\text{3}}\left({\text{X}}_{\text{t}}\right)=\{\begin{array}{c} 1:Xt=S4\\ 0.5:Xt=N1,N3\\ -1:Xt=(N4)\;or\;(S1,S3) \end{array}$$

$${\text{f}}_{\text{4}}\left({\text{X}}_{\text{t}}\right)=\{\begin{array}{c} 1:Xt=S4\\ -1:Xt=(N1,N3,N4)\;or\;(S1,S3) \end{array}$$

$${\text{f}}_{\text{5}}\left({\text{X}}_{\text{t}}\right)=\{\begin{array}{c} 1:Xt=S1,S3,S4\\ 0:Xt=N1,N3,N4 \end{array}$$

$${\text{f}}_{\text{6}}\left({\text{X}}_{\text{t}}\right)=\{\begin{array}{c} 1:Xt=S1,S3\\ -1:Xt=S4,N1,N3,N4 \end{array},$$

S: strong ball, N: normal ball (i.e., S4 means strong ball to P4).

$$\text{react}\left(\text{t}\right)=\{\begin{array}{c} 1:only\;session\;3\\ 0:other\;sessions \end{array}$$

$$\text{conf}\left(\text{t}\right)=\{\begin{array}{c} 0:sessions\;1,2,3\\ 1:session\;4\\ 2:session\;5,6,7,8 \end{array}$$

$$\text{message}\left(\text{t}\right)=\{\begin{array}{c} 0:sessions\;1,2,3,4,5\\ 1:sessions\;6,7,8 \end{array},$$

total_strong_balls(t): total number of strong balls from all players from the beginning of the task to trial t.

change(t)={0:sessions 1,2,3,4,51:sessions 6,7,8,if(S4 > 0 in session 4 or 5)and (S1,S3 > 0 in session 6,7,8)else 0:session 6,7,8.

U(X_t_) (X_t_ is N1, N3, N4, S1, S3, or S4; for instance, N1 stands for a normal ball to P1) contains seven linear coefficients (parameters β0, β1, β2, β3, β4, β5, and β6), which represent the contributions of different factors on bystander participation in interpersonal aggression. Because we need to distinguish ball throws to P4 from those to P1 or P3 due to their different meanings, X_t_ takes four values (N1 = N3, N4, S1 = S3 and S4).

When β1 is large, the participant tends to throw strong balls over all sessions independent of the session number or any specific context (see the definition of f_1_ above). β2 quantifies whether the participant seeks revenge to strong balls in session three or not (Dollard et al., 1939; Berkowitz, 1962). react(t) takes value 1 only in session 3, which is when participants receive strong balls from P4. β3 quantifies how closely the participant conforms to the interpersonal aggression of P1 and P3. conf(t) represents the strength of pressure to conform. From sessions 1–3, conf(t) takes a value of 0; in session 4, conf(t) is 1, since only P1 throws strong balls to P4; and from session 5 onward, conf(t) becomes 2, since both P1 and P3 throw strong balls to P4. When participants throw a normal ball to P1 or P3, f_3_(t) takes a value of 0.5, because normal balls to P1 or P3 assist the bullying to P4 indirectly. β4 quantifies how much a participant contributed to the aggression in response to the threatening message. We defined message(t) as the strength of the threat. In sessions 1–5, message(t) takes a value of 0; in sessions 6–8, message(t) becomes 1, since participants receive booster messages in sessions 6 and 7. β5 represents the effect of previous strong balls, in which total_strong_balls(t) is the total number of strong balls by all players from the beginning of the task to trial t. β5 also reflects the session effect. Most importantly for the present study, β6 was introduced to represent the effect of target-changing behavior. If a participant throws strong balls to P4 in sessions 4 or 5 and to P1 or P3 in sessions 6, 7, or 8, change(t) takes a value of 1. In all other conditions, change(t) takes a value of 0.

Before estimating the parameters, we normalized ball tossing scores to *z* scores in the preprocessing. Then, we estimated the seven parameters (β0 to β6; denoted as vectorθ) for each participant from their ball throws, X_t_, by the maximum likelihood estimation method of U(*X_t_*). Therefore, the minimization procedure of the negative log-likelihood of the participant’s behavior (D; i.e., a set of Xt) is identical to the multinomial logit model (Train, 2009), as shown in Equations 2 – 4. In Equation 2, β is a free parameter known as the inverse temperature parameter or slope and is determined by the maximum likelihood estimation. β1 in Equation 1 represents the bias toward a strong or normal ball. The nonlinear minimization of the negative log-likelihood was conducted by a standard technique (Daw, 2011) using the MATLAB function “fmincon.”

(2) $$\text{P}\left(Xt\right)=\frac{\text{exp}(\beta \cdot U\left(Xt\right))}{\sum_{Xc=N1,N3,N4}^{S1,S3,S4}\text{exp}(\beta \cdot U\left(Xc\right))},$$

(3) min (– log P (D |θ)),

(4) $$\text{P}(\text{D}\;|\theta )=\prod_{t}P\left(Xt\right).$$

### Questionnaire about past experiences of bullying

We used a questionnaire developed by the National Institute for Educational Policy Research of the Japanese Ministry of Education, Culture, Sports, Science, and Technology and widely used as the standard measure of bullying across Japan (Konishi et al., 2009). We asked the following questions about each participant’s experience of bullying over their school years. Binary points were assigned as follows: Yes = 1, No = 0. This questionnaire includes similar questions as other widely used and validated measures of bullying involvement (Austin and Joseph, 1996; Bosworth et al., 1999).

Items related to bullying (total: six points):

(1). I would ignore or scold my peers.
(2). I would say bad or threatening things.
(3). I would lightly push.
(4). I would hit or kick.
(5). I took money and other possessions.
(6). I harassed through computers or mobile phones.

Items related to victimhood (total: six points)

(1). I felt excluded, ignored, or shamed from social groups.
(2). I was teased or spoken badly of.
(3). I was hit lightly hit or kicked during play.
(4). I was physically harassed.
(5). I had my money taken or possessions broken.
(6). I was harassed through messages on my computer or smartphone.

We summed the points and calculated the two scales separately (bully and victim): the average bullying score was 1.744, SD = 1.399, range = 0–5; and the average victim score was 2.744, SD = 1.727, range = 0–6. We standardized scores for all participants (i.e., the mean score of participants = 0, SD = 1). A positive score on the scale designated the participant a bully or victim, and a participant could be designated both (bully/victim). As a result, among the 43 participants, we identified seven bullies, six victims, and 18 bully/victim, and 12 had no experience in bullying.

We conducted two other questionnaires for personality traits, the BFI (John and Srivastava, 1999) and the IRI (Davis, 1983; for replication purposes). The BFI consists of 70 items that measure the big five factors (dimensions) of personality: extraversion, agreeableness, neuroticism, openness, and intelligence. The IRI consists of 28 items answered on a five-point Likert scale. The measure has four subscales: perspective taking (P score), which measures the tendency to spontaneously adopt another’s point of view; fantasy (F score), which measures the tendency of the subject to shift themselves imaginatively to the feelings and actions of fictitious characters in books, movies, and plays; empathic concern (E score), which assesses other-oriented feelings of sympathy and concern for unfortunate others; and personal distress (D score), which assesses the feelings of anxiety and unease in tense interpersonal settings.

### fMRI data processing

Structural and resting-state functional MRI scans were performed using a 3T (Siemens Magnetom Trio A Tim System) MRI scanner at the Center for Information and Neural Networks (CiNet), National Institute of Information and Communications Technology with a 32-channel head coil. Functional images were acquired with a gradient echoplanar imaging (EPI) sequence of T2*-weighted images (repetition time (TR): 2500; echo time (TE): 30; flip angle: 90; field of view (FOV): 192 mm; voxel size: 3.0× 3.0 × 3.0 mm) during an 8-min rest condition, during which time participants were instructed to keep their eyes open and fixate. In addition, a high-resolution (1.0 × 1.0× 1.0 mm) structural scan was acquired from each participant with a T1-weighted MPRAGE sequence.

Although many studies have used an atlas-based definition, such as the Broadmann-based AAL (Uylings et al., 2005; Achard et al., 2006), this definition may not represent any of the constituent time courses if different functional areas are included within a single node. The Shen’s regions of interest (ROIs) we used were data-driven functional ROIs produced from the resting-state fMRI data of 79 healthy participants and parcellated by group-wise graph theory-based analysis (https://www.nitrc.org/frs/?group_id=51; Functional Brain Atlas from Shen et al., 2013). Similar to our previous study (Takami and Haruno, 2019), we focused on brain structures related to decision-making and emotion and excluded sensory, motor, and visual cortices (i.e., the cerebellum, visual, auditory, motor, and somatosensory areas) from the ROIs. Because Shen’s ROIs did not separate the amygdala, we adopted more finely divided definitions of the amygdala [i.e., amygdalastriatal (AStr), centro-medial (CM), latero-basal (LB), and superficial (SF)], which were taken from the SPM Anatomy toolbox (http://www.fz-juelich.de/inm/inm-1/DE/Forschung/_docs/SPMAnatomyToolbox/SPMAnatomyToolbox_node.html; see also Extended Data Figure 4-1). Thus, in total, we adopted 146 ROIs in our study: eight amygdala ROIs taken from the SPM Anatomy toolbox and 138 ROIs taken from Shen’s ROIs.

**Figure 4.**
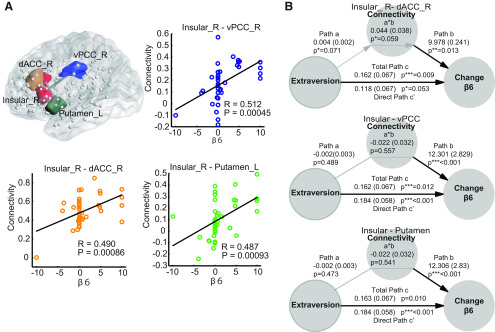
Resting-state fMRI results. Details of the ROIs are listed in Extended Data Figure 4-1. ***A***, Correlation of brain network links with behavioral parameters and personality traits. β6 (target-changer) had a significantly positive (*p* < 0.001) correlation with three brain network links (i.e., insula-PCC, insula-dACC, and insula-putamen). Edges in the brain illustrate these links. A significant correlation between the amygdala-TPJ connectivity and β3 is illustrated in Extended Data Figure 4-2. ***B***, Mediation analysis of the functional connectivity on the link from extraversion to β6. Path coefficients are shown next to arrows with standard errors in parentheses. Paths a and b represent the relationship of extraversion to connectivity, and from connectivity to β6 while controlling for extraversion. Path c’ represents the direct relationship from extraversion to β6 controlling for connectivity, and c represents the total relationship of extraversion to β6 (not adjusted for any other factors); ***p < 0.001, **p < 0.01, *p < 0.05, two-tailed. Black and gray arrows show significant (*p* < 0.05) and marginally significant (0.05 <*p* < 0.1) relationships, respectively.

10.1523/ENEURO.0273-19.2020.f4-1 Extended Data Figure 4-1 The 146 ROIs and MNI coordinates used for the resting-state MRI analysis. Eight amygdala subregions were adopted from the SPM anatomy toolbox and the other 138 ROIs were taken from Shen’s ROIs. Each ROI constitutes a gray ball depicted in Figure 4*A*. Download Figure 4-1, DOCX file.

**Figure 2.**
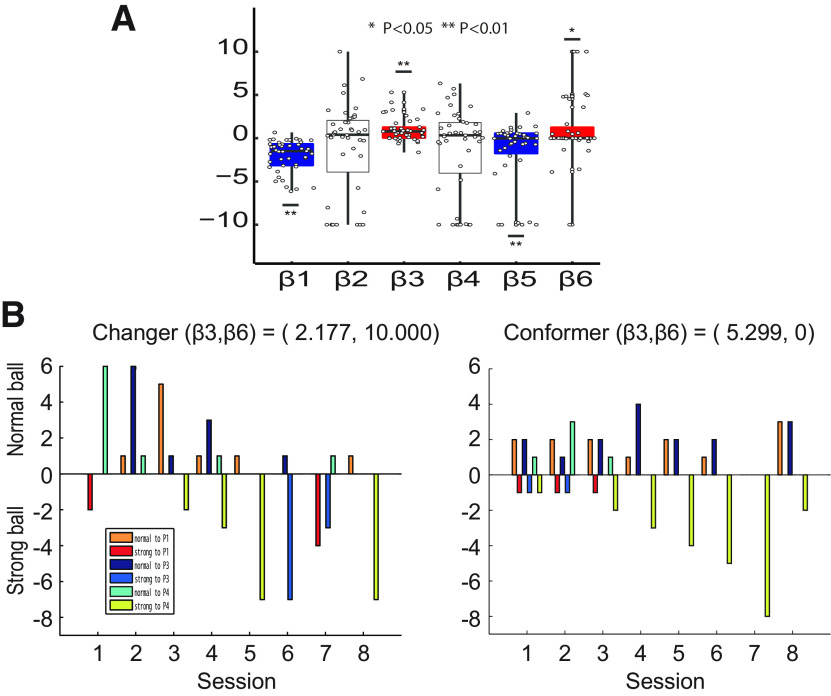
***A***, Estimates of β1, β2, β3, β4, β5, and β6. In boxplots, black lines within the box represent the median, and the edges of the box represent the 25th and 75th percentiles of the data. Dots and whiskers display all data points and their range. β3 and β6 distribute in the significantly positive area (red boxes), while β1 and β4 in the significantly negative area (blue boxes). ***B***, The behaviors of two players per session: one a target-changer with a high β6 value (left), and one a conformer with a high β3 value (right). In general, strong balls thrown by the target-changer to P3 and P1 increased between sessions 6 and 7, and strong balls thrown by the conformer to P4 increased after session 4.

10.1523/ENEURO.0273-19.2020.f4-2 Extended Data Figure 4-2 Correlation between β3 and amygdala-rTPJ connectivity. This result replicates our previous study (Takami and Haruno, 2019). Download Figure 4-2, EPS file.

Functional connectivity was analyzed with ROI-to-ROI correlation mapping using the CONN toolbox 18.a (http://web.conn-toolbox.org) based on SPM (Wellcome Department of Imaging Neuroscience, London, United Kingdom), since the removal of artefacts is a crucial first step in resting-state fMRI analysis. Spatial preprocessing of the CONN toolbox included realignment, normalization and smoothing (8-mm FWHM Gaussian filter) using SPM12 default parameter settings. Anatomical volumes were segmented into gray matter, white matter and CSF areas, and the resulting masks were eroded to minimize partial volume effects. The temporal time series characterizing the estimated subject motion (three-rotation and three-translation parameters, another six parameters representing the first-order temporal derivatives of these parameters, and scrubbing parameters containing the offending scans), as well as the BOLD time series within the subject-specific white matter mask [three principal component analysis (PCA) parameters] and the CSF mask (three PCA parameters) were used as temporal covariates and removed from the BOLD functional data using linear regression. The resulting residual BOLD time series were then bandpass filtered (0.008 Hz < f < 0.10 Hz).

Pearson correlation coefficients between the time courses of each possible pair of nodes were calculated and normalized to *z* scores using the Fisher transformation, resulting in a 146 × 146 symmetrical connectivity matrix for each participant (ROI-to-ROI analysis in CONN). We used MATLAB for this procedure.

The next step was to select an informative set of connectivity to predict the behavioral β values β6 and β3 from the elements of the connectivity matrix. For this analysis, we used LASSO (least absolute shrinkage selection operator; Tibshirani, 1996) in the R package glmnet. More specifically, we conducted 10-fold cross-validation for selecting the optimal value of λ that gave a minimum mean cross-validated error by the cv.glmnet function in the glmnet R package. Using this optimal λ value, we identified which of the computed 146 × 146 edges contributed to the predictions of β6 and β3. We then evaluated the significance of the correlation between the selected set of connectivity and β6 and β3. This predictive feature selection-based method increased the sensitivity of multivariate regressions (Pereira et al., 2009). To visualize brain network links, we used BrainNet Viewer (Xia et al., 2013; http://www.nitrc.org/projects/bnv/).

### Mediation analysis

To quantify and test whether resting state functional connectivity mediates an effect of extraversion on behavioral effects (target-change, or β6 in the present study), we performed a standard mediation analysis using a mediation tool box (https://github.com/canlab/MediationToolbox; Wager et al., 2008). This analysis quantifies in general the degree to which a relationship between two variables, X and Y, can be explained by another variable, M. We defined X as personality trait scores, Y as a behavioral parameter, and M as resting state connectivity (Fig. 4*B*).

Paths a and b in Figure 4*B* measure the association between personality trait scores and the mediator (resting state connectivity) and the association between the mediator and behavioral parameter while controlling for personality trait scores, respectively. More specifically, path b tests whether resting state connectivity predicts variations in the behavioral parameter that are conditionally independent of the personality trait scores.

On the other hand, paths c and c’, respectively, measure the total relationship between the personality trait scores and behavioral parameter including direct and indirect effects and the direct effect of the relationship between the personality trait scores and behavioral parameter while controlling for resting state connectivity. Finally, product a*b tests the significance of the mediators. We conducted bootstrap tests (10,000 iterations) to determine statistical significance of the mediators.

## Results

### Basic behavioral results

Figure 1*B* shows the mean frequency of participants’ strong and normal ball throws. The positive region shows the means of normal balls, and the negative region shows the means of strong balls. We conducted a repeated-measure analysis of variance to the number of strong balls thrown to P1 and P3 over all eight sessions. There were significant effects of sessions for both P1 (*F*_(7,336)_ = 3.460, *p* = 0.001) and P3 (*F*_(7,336)_ = 4.472, *p* < 0.001). Furthermore in session 6, in which participants received a boost message from P3, strong balls thrown to P3 significantly increased compared with session 5 (*t*_(42)_ = 6.110, *p* < 0.0001, paired *t* test). Similarly, in session 7, in which participants received a threat message from P1, strong balls thrown to P1 significantly increased (*t*_(42)_ = 2.565, *p* = 0.014, paired *t* test). Thus, the task was able to produce target-changing behaviors.

In order to investigate this behavior in more detail, we extended the model-based analysis used in our previous report and incorporated a term representing target-changing behaviors (i.e., β6). Therefore, our computational model included six parameters in the utility function: β1 (baseline propensity for aggression), β2 (reactive revenge), β3 (conformity to aggression), β4 (capitulation to threat), β5 (effect of previous strong balls), and β6 (target-changing). In addition to these, we also included β (slope in Eq. 2; see also Materials and Methods). We estimated these parameters for each participant by the maximum likelihood estimation method based on P2 (participant) ball throws to P1, P3, and P4.

We found that only β3 and β6 were significantly positive, while β1 and β4 were significantly negative (one sample *t* test, β1; *t*_(42)_ = −7.205, *p* < 0.0001, β2; *t*_(42)_ = −1.359, *p* = 0.182, β3; *t*_(42)_ = 4.774, *p* < 0.0001, β4; *t*_(42)_ = −1.634, *p* = 0.110, β5; *t*_(42)_ = −2.907, *p* < 0.01 and β6; *t*_(42)_ = 2.042, *p* < 0.05; Fig. 2*A*). These results demonstrate that conformity and target-changing are main driving forces in peer-influenced bystander participation in interpersonal aggression (β3 and β6), and also that a participant’s default action is a normal ball (β1) and that a participant does not participate in aggression in response to a threat (β4). In this analysis, the estimated values of β (slope; mean = 0.770, SD = 1.111) were comparable among participants. Figure 2*B* exemplifies behaviors of typical participants. For a target-changer who showed a high β6 (β3 = 2.177, β6 = 10.000; Fig. 2*B*, left), the number of strong balls thrown to P3 in session 6 increased sharply, and strong balls thrown to P1 increased in session 7. On the other hand, for a conformer who exhibited a high β3 (β3 = 5.299, β6 = 0; Fig. 2*B*, right), the number of strong balls thrown to P4 gradually increased from sessions 3–6.

**Figure 3.**
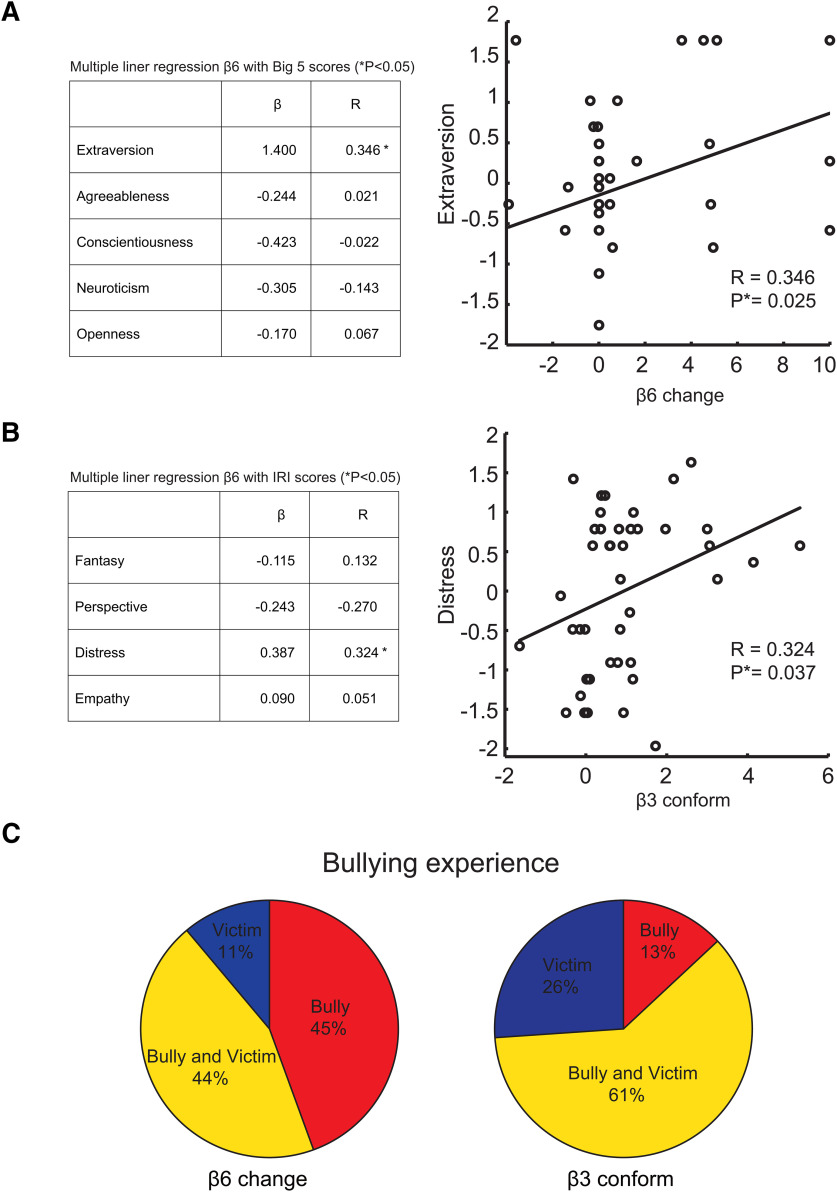
***A***, Correlation of Big5 scores with β6 (target-changer). Left, Multiple linear regression of β6 using Big5 scores (*p < 0.05). Right, β6 had a positive correlation with extraversion (one outlier point excluded). ***B***, Correlation of IRI scores with β3 (conformity). Left, Multiple linear regression of β3 using IRI scores (*p < 0.05). β3 had a positive correlation with personal distress (one outlier point excluded). ***C***, Pie graphs show the percentage of bullying experiences for participants who had a positive effect of β6 (left) and β3 (right). Red, yellow, and blue represent experiences as bully only, both bully and victim, and victim only, respectively. In β6-positive participants (left), the percentage of bully only was 45%. In β3-positive participants (right), the percentage of victim only was higher than that of β6-positive participants.

We then examined whether personality trait scores are correlated with target-changing and conformity to aggression (i.e., β6 and β3). We first conducted multiple linear regressions based on all scores in BFI and IRI separately (Fig. 3*A*, left, *B*, left). We found that only Extraversion in the BFI and Personal Distress in the IRI had a significant (positive) effect on β6 (target-changing) and β3 (conformity to bullying), respectively. We also found a significant correlation between extraversion and β6 (*R* = 0.346, *p* = 0.025; Fig. 3*A*, right) and between personal distress and β3 (R = 0.324, *p* = 0.037; Fig. 3*B*, right), which is consistent with our previous report (Takami and Haruno, 2019).

We next considered whether β6 and β3 have potential links with the real-world bullying experiences of the participants. Figure 3*C* shows that 45% of participants who had positive β6 had bully-only experiences, while the percentage of participants who had victim-only experiences tended to show positive β3, suggesting that target-changing and conformity to aggression may be linked more tightly with bully and victim experiences, respectively. These results, combined with those seen in Figure 3*B*, are consistent with many previous reports that found victims tend to have higher anxiety (Slee, 1994; Espelage and Holt, 2001; Swearer et al., 2001).

We calculated ROI-to-ROI functional connectivity using the CONN tool box and 146 ROIs (Extended Data Fig. 4-1) based on the state-of-the-art Functional Brain Atlas (Shen et al., 2013), which is constructed from healthy-population resting-state fMRI data (see Materials and Methods). Pearson correlation coefficients between the time courses of each possible pair of nodes were calculated and used to construct 146 × 146 symmetrical connectivity matrices, where each element defines the connection strength of an edge between two nodes. A connectivity matrix was constructed for each participant, and the Pearson correlation coefficients between the elements of the matrix and the corresponding participant’s behavioral parameter β3 (conform to bullying) and β6 (target-changing) were computed. To avoid overfitting to high dimensional components of the connectivity matrix, we first conducted a feature selection by using LASSO (see Materials and Methods) with the 10-fold cross-validation method to identify which of the computed 146 × 146 edges contributes to the prediction of β6 and β3. As a result, we found 46 and 32 edges for β6 and β3, respectively. Finally, to evaluate the significance of the correlation coefficients between these selected edges and β6 and β3, we set the statistical threshold to Bonferroni corrected *p* < 0.05 for the number of edges (equivalent to uncorrected *p* < 0.0001).

We found three links significantly and positively correlated with β6 (target-changing): connections between the right insular cortex and the ventral PCC (vPCC_R; *p* = 0.00045 uncorrected and *R* = 0.512), the right insular cortex and the right dACC (dACC_R; *p* = 0.00086 and *R* = 0.490), and the right insular cortex and the left putamen (*p* = 0.00093 uncorrected and *R* = 0.487; Fig. 4*A*; Table 1). We also found that the connectivity between the left amygdala [MNI −21 −6 −14] and right TPJ [MNI 55 −17 −3] were correlated with β3 (conform to aggression; Extended Data Fig. 4-2), again consistent with our previous report (Takami and Haruno, 2019).

**Table 1:** Brain connectivity strength that correlated with β6 (target-change; *p* < 0.001)

Node	MNI			Node	MNI			*R*	*p*
Insula_R	34	15	–6	vPCC_R	7	–18	30	0.512	0.00045
Insula_R	34	15	–6	dACC_R	7	30	17	0.490	0.00086
Insula_R	35	19	8	Putamen_L	–10	4	–6	0.487	0.00093

We also conducted a mediation analysis, as it is important to examine whether the relationship between personality trait score (extraversion) and behavior (β6, i.e., target-changing) is mediated by these three connectivity links including the insula, as shown in Figure 4*A*. As a result, only the connectivity between the insula and dACC was a marginally significant mediator from extraversion to β6 (Fig. 4*B*; the coefficient for path c = 0.162, z = 2.611, *p* = 0.038). We also found the functional connectivity was significantly associated with β6 even after controlling for extraversion (the coefficient for path b = 9.978, z = 2.480, *p* = 0.241). At the same time, extraversion was marginally associated with the connectivity (the coefficient for path a = 0.004, z = 1.806, *p* = 0.071), with a marginally significant mediation effect of the connectivity (a*b coefficient = 0.044, z = 1.885, *p* = 0.059). These data suggest that increased extraversion is associated with more target-changing behavior through the effect of the connectivity between the insula and dACC.

## Discussion

In this study, we observed that a majority of participants in the catch-ball task initially conformed to two other players (P1 and P3; Fig. 2*B*) who threw strong balls to another player (P4), but some of these conformers later changed their target to P1 and P3 (target-change). To investigate this target-changing behavior, we extended our prior model analysis and found that target-changing as well as conformity to aggression had a significantly positive effect on interpersonal aggression. Furthermore, there was a correlation between a participant’s target-changing and extraversion, and between conformity to aggression and personal distress (i.e., social anxiety and unease). A questionnaire about past experiences of bullying revealed that bullies and victims are more involved in target-changing and conforming to aggression, respectively. Our resting-state fMRI analysis demonstrated that three links involving the insula, dACC, vPCC, and putamen had a significant correlation with target-changing behavior. We also found that only insular-dACC connectivity partially mediated the relationship between extraversion and target-changing.

It was intriguing that target-changers exhibited high extraversion according to the BFI (Fig. 3*A*), which is characterized by excitability, sociability, assertiveness, and high amounts of emotional expressiveness (John and Srivastava, 1999). Extraversion has been suggested to have two important implications for the emergence of social networks: the popularity effect assumes extraverts gather more friends than introverts, and the homophile effect assumes the more similar two people’s levels of extraversion the more likely they are to become friends. Related to these two effects, many previous studies documented that bullies are popular or stylish (Graham et al., 1992; Parkhurst and Hopmeyer, 1998; Gest et al., 2001; LaFontana and Cillessen, 2001; Vaillancourt et al., 2003) and that bullies tend to be extraverts (Connolly and O'Moore, 2003; Tani et al., 2003). It was also reported that aggressive youths are often perceived as “popular” by peers (Rodkin et al., 2000; LaFontana and Cillessen, 2002), and perceived popularity is associated with both prosocial and aggressive behaviors (Luthar and McMahon, 1996; Parkhurst and Hopmeyer, 1998; Rodkin et al., 2000). Another study reported that perceived popularity correlated with relational aggression among older youths, but not with overt aggression (Rose et al., 2004).These observations are in good agreement with our evolutionary view that target-changing is a form of bistrategic (i.e., prosocial and coercive) behavior as advocated in the resource control theory (Hawley, 2002, 2003; Little et al., 2007), in which target-changing promotes the social adaptability of aggression by displaying prosocial intention (i.e., helping a victim or punishing a bully) of attack to other people explicitly and helping to gain popularity from them.

The bistrategic view of target-changing can also be linked with previous fMRI studies of human prosociality, which found rejecting unfair offers comprises automatic intuition and context-dependent reflection (Haruno and Frith, 2010; Haruno et al., 2014). Automatic intuition is mainly supported by subcortical structures such as the amygdala and striatum, whereas context-dependent reflection is mainly supported by cortical structures such as the dACC, insula, and dorsolateral prefrontal cortex. The context-dependent nature of target-changing suggests that the dACC, insula, and dorsolateral prefrontal cortex also play a key role in target-changing.

Our resting-state fMRI results suggested that connectivity between the insula and dACC is pivotal to target-changing behavior. The insular cortex has been implicated in processing disgust (Phillips et al., 1997; Krolak-Salmon et al., 2003; Wright et al., 2004). Our task setting receiving message (“Let’s throw more strong balls to P4”) in session 6 might evoke such an emotion in the insular cortex. It is also reported that activity in the dACC reflects foraging (Kolling et al., 2012; Shenhav et al., 2013) and adaptive task-switching behaviors (MacDonald et al., 2000; De Baene and Brass, 2013; Economides et al., 2014; von der Gablentz et al., 2015; Sarafyazd and Jazayeri, 2019). In addition, previous resting-state MRI studies established that the insula and dACC form a “salience network” that facilitates the detection of important environmental stimuli (Menon and Uddin, 2010; Cauda et al., 2011). Altogether, it is plausible that insula-dACC connectivity works in a concerted manner to decide whether an individual continues to present aggression or change the target based on his emotional state. We also showed that only the connectivity between the insula and dACC mediated the relationship between extraversion and target-changing behavior. Consistently, previous studies have reported that extraversion is related to the dACC, insula and amygdala (Eisenberger et al., 2005; Aghajani et al., 2014; Lei et al., 2015).

There are several limitations to the present study. First, the motivation behind target-changing seems to be mixed in the present design of the task. More specifically, some participants may change the target (P4) to P1 and P3 for aggressive purposes, while others may do so for prosocial purposes to help P4 or punish P1 and P3 (Fehr and Gächter, 2002). However, it is also possible that this duality of motivation is the essence of target-changing as discussed above from the bistrategic view of aggression. A second limitation is that only a small number of bullies and victims were identified in the questionnaire about past experiences of bullying, compromising the reliability of the results linking task behaviors and real-world bullying. It would also be necessary in future studies to use bullying questionnaires with more specific time frames (e.g., within recent years) and to disentangle those who bully from those who are simply more aggressive generally (Hawley et al., 2011; Ybarra et al., 2014). Finally, a third limitation of the present study is the reliance on resting-state fMRI for the neural correlates of interpersonal aggression. To overcome this limitation, future studies would require a novel task design that allows us to conduct task fMRI experiments with a higher level of reality.

Despite these limitations, this study revealed that target-changing and conforming behaviors in interpersonal aggression have dissociable behavioral and neural mechanisms, and also suggested that these two processes are differently involved in real-world interpersonal aggression. Our results are also consistent with the bistrategic (simultaneously prosocial and coercive) view of target-changing behavior. These contributions were made possible by a model-based integration of behaviors during a novel catch-ball task, questionnaires about past experiences of bullying, personality trait scores and resting-state fMRI.

## Synthesis

Reviewing Editor: Philippe Tobler, University of Zurich Decisions are customarily a result of the Reviewing Editor and the peer reviewers coming together and discussing their recommendations until a consensus is reached. When revisions are invited, a fact-based synthesis statement explaining their decision and outlining what is needed to prepare a revision will be listed below.

Your work has now been seen again by *the two* reviewers (please note that it is journal policy to combine the separate reviews into a single synopsis, which both reviewers have seen and could/did comment on).

The reviewers agreed that you took significant steps in addressing the concerns raised previously. However , some concerns remained. These concern primarily conceptual integration of the study into the previous literature and the statistics.

Major concerns

1) There continues to be a lack of framing this study in the larger context of the literature of examining aggression and bullying, and prior attempts to study these phenomenon via neuroscience. There is still a lack of connection of the present work to the larger literature of bullying/aggression, and to the few neuroscience studies that connect to this work. The way the paper is currently written, there are no strong connections made by the authors to much theory about the behavior that seemingly motivated the study. Please address this point carefully by really going into the literature and working out the relation between your study and what has been done before.

2) Relatedly, where the authors did incorporate this literature, it is not always well integrated into the manuscript.

For example, the authors write: “social adaptability of overt aggression [unclear what that means] by rationalizing the attack explicitly to others” - this is jargony, implies cognition and stated motivational purposes, and it is unclear what it means. It is used twice in the manuscript and needs to be written more clearly. (below are the other portions of text where this phrase was stated)

a. "Often in bullying, the victim changes (target-changing). From an evolutionary point of view (Hawley, 2002, 2003; Hawley et al., 2007; Hawley et al., 2011), target-changing behavior may have some evolutionary advantage. For instance, it may increase the social adaptability of overt aggression by rationalizing the attack explicitly to others (Kaukiainen et al., 1999; Rose et al., 2004).” -

b. "These observations seem to be consistent with our evolutionary view of target-changing behaviors, in which target-changing may promote the social adaptability of overt aggression by displaying the reason of attack to other people explicitly and helping to gain popularity from them (Kaukiainen et al., 1999; Rose et al., 2004).”

3) The authors still do not seem to have provided unstandardized raw scores from the bullying and victimization questionnaires within the sample. At a minimum, they should include that in the text, i.e. the average bullying score was x.xx, sd=xx, range=0-XX, same with victimization. This way, we know how to compare the scores IN THIS STUDY to results from other studies. Given that bullies were determined by a “greater than 0” standardized score, it is essential to know the range of bullying scores in the sample for interpretation purposes

4) It is unclear which tuning parameter the authors used for LASSO regression, and how they decided upon a particular value. Usually this is done with cross-validation, but their sample size is quite small, so there is a concern that they may have either used a potentially inappropriate default or ended using a value that is inadequate.

5) It appears the authors ‘peeked’ at their data and ran pairwise comparisons after having seen the cell means in a prior version of the manuscript. At this point, they should use Tukey’s HSD. In other words, they’re adjusting their comparisons after having seen the data, it is inappropriate to correct using Bonferroni as if they only tested a subset of possible comparisons.

6) It is still unclear how the ROIs were defined. The authors appear to be using some kind of atlas? But one remains unsure. This needs to be made more explicit in order to better gauge the validity of the results.

7) The authors can still take steps to standardize statistical reporting - three decimal places rather than interchanging between three and four - throughout, not just in this section: “The positive region shows the means of normal balls, and the negative region shows the means of strong balls. We conducted a repeated-measure ANOVA to the number of strong balls thrown to P1 and P3 over all 8 sessions. There were significant effects of sessions for both P1 (F (7,336) =3.46, P=0.0014)) and P3 (F (7,336) =4.47, P<0.001). Furthermore in session 6, in which participants received a boost message from P3, strong balls thrown to P3 significantly increased compared with session 5 (t(42)=6.1102,P<0.0001, paired t-test). Similarly, in session 7, in which participants received a threat message from P1, strong balls thrown to P1 significantly increased (t (42) =2.5652, P＝0.0140, paired t-test). Thus, the task was able to produce target-changing behaviors.

Minor suggestions for problematic grammar/syntax

1) "Actors in interpersonal aggression, such as bullies, change their targets frequently, but the underlying behavioral and neural mechanisms are unknown.”

2) "Here, using the catch-ball task we recently developed to examine interpersonal aggression, we found that target-changing and conforming to other participants’ aggression were major driving forces of increased aggression (i.e., throwing strong balls). Additionally, we found that these two behaviors were correlated with a participant’s extraversion and social anxiety, respectively. In addition, questionnaires about participants' past experiences of bullying suggested that target-changers and conformers were predominantly bullies and victims in the past. An analysis of resting-state fMRI revealed that functional connectivity between the dorsal anterior cingulate cortex and insula were correlated with target-changing behavior, while functional connectivity between the amygdala and temporo-parietal junction was correlated with conformity. These results indicate that target-changing and conforming behaviors have dissociable behavioral and neural mechanisms and may contribute to real-world interpersonal aggressions differently.”

3) "To link the results obtained from these experiments to real-world interpersonal aggression and the personality traits of the participants, we included a self-report measure of past experiences of bullying, a self-report measure of personality (Big Five Inventory:BFI, John and Srivastava, 1999) and a self-report measure of empathy (Interpersonal Reactivity Index: IRI, Davis, 1983). We hypothesized that the dorsal anterior cingulate cortex (dACC) may play an important role, because this brain structure was revealed to correlate with conformity to interpersonal aggression in our previous study (Anonymous, 2019), with social exclusion in a similar catch ball setting (i.e., cyber-ball game) (Kawamoto et al., 2012), and with behavioral switching (Kolling et al., 2012).”

4) "To quantify and test whether resting state functional connectivity mediates effect of extraversion on behavioral effects (target change, or β6 in the present study), we performed a standard mediation analysis (https://github.com/canlab/MediationToolbox) (Wager et al., 2008).”

5) "One may argue that the participants might have noticed the programmed nature of the computer players. Our post-experiment questionnaire revealed that almost 90% of participants did not have this suspicion (see our previous report (Anonymous 2019) for details)” ought to be changed to “We included a post-experiment questionnaire to assess the ecological validity of the task, (∼90% accepted the task manipulation as valid, see Anonymous 2019 for further details).”
