# Supplementary material for: Dissociable Behavioral and Neural Correlates for Target-Changing and Conforming Behaviors in Interpersonal Aggression
Source: eNeuro. 2020 Jun 1;7(3):ENEURO.0273-19.2020. doi: 10.1523/ENEURO.0273-19.2020 (PMC7294470; doi:10.1523/ENEURO.0273-19.2020)
Supplement: Extended Data Figure 4-1 — The 146 ROIs and MNI coordinates used for the resting-state MRI analysis. Eight amygdala subregions were adopted from the SPM anatomy toolbox and the other 138 ROIs were taken from Shen’s ROIs. Each ROI constitutes a gray ball depicted in Figure 4A. Download Figure 4-1, DOCX file. [file enu-eN-NWR-0273-19-s02.docx]

|  | ROI | MNI |  |  |
| --- | --- | --- | --- | --- |
| 1 | Dorsolateral_Prefrontal_Cortex_L | -45 | 26 | 24 |
| 2 | Dorsolateral_Prefrontal_Cortex_L | -27 | 36 | 31 |
| 3 | Dorsolateral_Prefrontal_Cortex_L | -10 | 47 | 36 |
| 4 | Anterior_Prefrontal_Cortex_L | -38 | 42 | 26 |
| 5 | Anterior_Prefrontal_Cortex_L | -32 | 50 | -1 |
| 6 | Anterior_Prefrontal_Cortex_L | -27 | 48 | 18 |
| 7 | Anterior_Prefrontal_Cortex_L | -16 | 60 | 3 |
| 8 | Anterior_Prefrontal_Cortex_L | -12 | 55 | -13 |
| 9 | Anterior_Prefrontal_Cortex_L | -10 | 57 | 20 |
| 10 | Anterior_Prefrontal_Cortex_L | -7 | 47 | 0 |
| 11 | Orbitofrontal_Area_L | -9 | 40 | -20 |
| 12 | Orbitofrontal_Area_L | -7 | 20 | -17 |
| 13 | Insular_Cortex_L | -39 | -11 | 13 |
| 14 | Insular_Cortex_L | -36 | 7 | -1 |
| 15 | Insular_Cortex_L | -35 | -12 | 0 |
| 16 | Inferior_Temporal_Gyrus_L | -49 | -11 | -26 |
| 17 | Inferior_Temporal_Gyrus_L | -57 | -43 | 7 |
| 18 | Inferior_Temporal_Gyrus_L | -56 | -12 | -10 |
| 19 | Inferior_Temporal_Gyrus_L | -56 | -29 | -13 |
| 20 | Inferior_Temporal_Gyrus_L | -54 | -27 | 3 |
| 21 | Superior_Temporal_Gyrus_L | -52 | 0 | 2 |
| 22 | Ventral_Posterior_Cingulate_Cortex_L | -8 | -56 | 21 |
| 23 | Ventral_Posterior_Cingulate_Cortex_L | -4 | -17 | 29 |
| 24 | Ventral_Posterior_Cingulate_Cortex_L | -4 | -33 | 32 |
| 25 | Ventral_Anterior_Cingulate_Cortex_L | -4 | 27 | 14 |
| 26 | Ventral_Anterior_Cingulate_Cortex_L | -4 | 4 | 24 |
| 27 | Ventral_Anterior_Cingulate_Cortex_L | -6 | -42 | 17 |
| 28 | Dorsal_Posterior_Cingulate_Cortex_L | -8 | -40 | 47 |
| 29 | Dorsal_Posterior_Cingulate_Cortex_L | -8 | -22 | 42 |
| 30 | Dorsal_Posterior_Cingulate_Cortex_L | -6 | -49 | 36 |
| 31 | Dorsal_Anterior_Cingulate_Cortex_L | -7 | 30 | 29 |
| 32 | Dorsal_Anterior_Cingulate_Cortex_L | -6 | 42 | 17 |
| 33 | Dorsal_Anterior_Cingulate_Cortex_L | -6 | 32 | -7 |
| 34 | Dorsal_Anterior_Cingulate_Cortex_L | -5 | 12 | 35 |
| 35 | Perirhinal_Cortex_L | -32 | -15 | -24 |
| 36 | Temporopolar_Area_L | -46 | 0 | -12 |
| 37 | Temporopolar_Area_L | -41 | 8 | -26 |
| 38 | Temporopolar_Area_L | -26 | 2 | -31 |
| 39 | Angular_Gyrus_L | -54 | -49 | 26 |
| 40 | Angular_Gyrus_L | -51 | -44 | 41 |
| 41 | Angular_Gyrus_L | -51 | -57 | 14 |
| 42 | Angular_Gyrus_L | -48 | -60 | 30 |
| 43 | Angular_Gyrus_L | -39 | -61 | 44 |
| 44 | Supramarginal_Gyrus_L | -57 | -28 | 33 |
| 45 | Supramarginal_Gyrus_L | -57 | -24 | 16 |
| 46 | Supramarginal_Gyrus_L | -55 | -38 | 23 |
| 47 | Supramarginal_Gyrus_L | -44 | -32 | 44 |
| 48 | Inferior_frontal_Gyrus_L | -52 | 15 | 14 |
| 49 | Inferior_frontal_Gyrus_L | -35 | 5 | 11 |
| 50 | Inferior_frontal_Gyrus_L | -51 | 29 | 6 |
| 51 | Inferior_frontal_Gyrus_L | -32 | 22 | 4 |
| 52 | Dorsolateral_Prefrontal_Cortex_L | -40 | 41 | 11 |
| 53 | Inferior_Frontal_Gyrus_L | -44 | 40 | -5 |
| 54 | Inferior_Frontal_Gyrus_L | -44 | 19 | -8 |
| 55 | Inferior_Frontal_Gyrus_L | -28 | 35 | -12 |
| 56 | Inferior_Frontal_Gyrus_L | -25 | 16 | -16 |
| 57 | Caudate_L | -14 | -15 | 20 |
| 58 | Caudate_L | -12 | 7 | 13 |
| 59 | Caudate_L | -11 | 18 | 2 |
| 60 | Putamen_L | -23 | 4 | 2 |
| 61 | Putamen_L | -10 | 4 | -6 |
| 62 | Thalamus_L | -18 | -35 | 15 |
| 63 | L.BA50 | -10 | -26 | 3 |
| 64 | ParaHippocampal_L | -3 | -10 | 8 |
| 65 | Amygdala_AStr_L (SPM Anatomy toolbox) | -27 | -7 | -15 |
| 66 | Amygdala_CM_L (SPM Anatomy toolbox) | -21 | -6 | -14 |
| 67 | Amygdala_LB_L (SPM Anatomy toolbox) | -23 | -2 | -24 |
| 68 | Amygdala_SF_L (SPM Anatomy toolbox) | -15 | -5 | -19 |
| 69 | Hippocampus_L | -28 | -20 | -9 |
| 70 | Hippocampus_L | -27 | -38 | -1 |
| 71 | BA55.1_L | -4 | -24 | -9 |
| 72 | Dorsolateral_Prefrontal_Cortex_R | 6 | 45 | 21 |
| 73 | Dorsolateral_Prefrontal_Cortex_R | 14 | 48 | 36 |
| 74 | Dorsolateral_Prefrontal_Cortex_R | 24 | 48 | 25 |
| 75 | Dorsolateral_Prefrontal_Cortex_R | 37 | 37 | 26 |
| 76 | Dorsolateral_Prefrontal_Cortex_R | 50 | 23 | 27 |
| 77 | Anterior_Prefrontal_Cortex_R | 8 | 42 | 2 |
| 78 | Anterior_Prefrontal_Cortex_R | 13 | 60 | 0 |
| 79 | Anterior_Prefrontal_Cortex_R | 13 | 58 | 17 |
| 80 | Anterior_Prefrontal_Cortex_R | 29 | 52 | -5 |
| 81 | Anterior_Prefrontal_Cortex_R | 32 | 49 | 12 |
| 82 | Anterior_Prefrontal_Cortex_R | 43 | 43 | -2 |
| 83 | Orbitofrontal_Area_R | 5 | 30 | -6 |
| 84 | Orbitofrontal_Area_R | 6 | 41 | -17 |
| 85 | Orbitofrontal_Area_R | 8 | 24 | -19 |
| 86 | Orbitofrontal_Area_R | 11 | 53 | -16 |
| 87 | Insular_Cortex_R | 34 | 15 | -6 |
| 88 | Insular_Cortex_R | 39 | 4 | 4 |
| 89 | Insular_Cortex_R | 40 | -6 | 14 |
| 90 | Insular_Cortex_R | 41 | -11 | 0 |
| 91 | Inferior_Temporal_Gyrus_R | 36 | -16 | -25 |
| 92 | Inferior_Temporal_Gyrus_R | 46 | 0 | -29 |
| 93 | Inferior_Temporal_Gyrus_R | 46 | -53 | -33 |
| 94 | Middle_Temporal_Gyrus_R | 50 | -32 | 3 |
| 95 | Middle_Temporal_Gyrus_R | 55 | -14 | -20 |
| 96 | Middle_Temporal_Gyrus_R | 58 | -29 | -12 |
| 97 | Middle_Temporal_Gyrus_R | 62 | -40 | 0 |
| 98 | Superior_Temporal_Gyrus_R | 55 | -17 | -3 |
| 99 | Superior_Temporal_Gyrus_R | 56 | 0 | 2 |
| 100 | Superior_Temporal_Gyrus_R | 62 | -29 | 12 |
| 101 | Ventral_Posterior_Cingulate_Cortex_R | 6 | -42 | 19 |
| 102 | Ventral_Posterior_Cingulate_Cortex_R | 6 | -39 | 37 |
| 103 | Ventral_Posterior_Cingulate_Cortex_R | 7 | -18 | 30 |
| 104 | Ventral_Posterior_Cingulate_Cortex_R | 12 | -55 | 16 |
| 105 | Ventral_Anterior_Cingulate_Cortex_R | 7 | -6 | 43 |
| 106 | Dorsal_Posterior_Cingulate_Cortex_R | 6 | -54 | 31 |
| 107 | Dorsal_Posterior_Cingulate_Cortex_R | 9 | -28 | 45 |
| 108 | Dorsal_Posterior_Cingulate_Cortex_R | 17 | -63 | 27 |
| 109 | Dorsal_Anterior_Cingulate_Cortex_R | 6 | 11 | 30 |
| 110 | Dorsal_Anterior_Cingulate_Cortex_R | 7 | 30 | 17 |
| 111 | Perirhinal_Cortex_R | 11 | -43 | -17 |
| 112 | Perirhinal_Cortex_R | 15 | -43 | 3 |
| 113 | Temporopolar_Area_R | 29 | 0 | -32 |
| 114 | Temporopolar_Area_R | 37 | 12 | -24 |
| 115 | Temporopolar_Area_R | 50 | 1 | -13 |
| 116 | Angular_Gyrus_R | 33 | -67 | 38 |
| 117 | Angular_Gyrus_R | 48 | -59 | 34 |
| 118 | Angular_Gyrus_R | 48 | -57 | 17 |
| 119 | Angular_Gyrus_R | 58 | -47 | 24 |
| 120 | Supramarginal_Gyrus_R | 48 | -29 | 18 |
| 121 | Supramarginal_Gyrus_R | 49 | -31 | 49 |
| 122 | Supramarginal_Gyrus_R | 51 | -46 | 40 |
| 123 | Supramarginal_Gyrus_R | 57 | -20 | 31 |
| 124 | Supramarginal_Gyrus_R | 58 | -34 | 30 |
| 125 | Inferior_frontal_Gyrus_R | 36 | 17 | 29 |
| 126 | Inferior_frontal_Gyrus_R | 52 | 13 | 12 |
| 127 | Inferior_frontal_Gyrus_R | 35 | 19 | 8 |
| 128 | Dorsolateral_Prefrontal_Cortex_R | 46 | 37 | 13 |
| 129 | Inferior_Frontal_Gyrus_R | 18 | 12 | -15 |
| 130 | Inferior_Frontal_Gyrus_R | 26 | 33 | -13 |
| 131 | Inferior_Frontal_Gyrus_R | 43 | 24 | -11 |
| 132 | Inferior_Frontal_Gyrus_R | 52 | 25 | 3 |
| 133 | Caudate_R | 12 | 17 | 4 |
| 134 | Caudate_R | 14 | 0 | 17 |
| 135 | Putamen_R | 24 | 5 | 1 |
| 136 | Putamen_R | 29 | -8 | -1 |
| 137 | Thalamus_R | 5 | -13 | 7 |
| 138 | R.BA50 | 8 | -25 | -7 |
| 139 | ParaHippocampal_R | 15 | -30 | 15 |
| 140 | Amygdala_AStr_R (SPM Anatomy toolbox) | 28 | -6 | -15 |
| 141 | Amygdala_CM_R (SPM Anatomy toolbox) | 23 | -5 | -14 |
| 142 | Amygdala_LB_R (SPM Anatomy toolbox) | 25 | -1 | -24 |
| 143 | Amygdala_SF_R (SPM Anatomy toolbox) | 18 | -4 | -18 |
| 144 | Hippocampus_R | 27 | -19 | -11 |
| 145 | Hippocampus_R | 30 | -36 | 0 |
| 146 | R.BA55.1_R | 8 | 1 | -4 |
